# Patient Reported Delays in Seeking Treatment for Tuberculosis among Adult and Pediatric TB Patients and TB Patients Co-Infected with HIV in Lima, Peru: A Qualitative Study

**DOI:** 10.3389/fpubh.2014.00281

**Published:** 2014-12-22

**Authors:** Valerie A. Paz-Soldan, Rebecca E. Alban, Christy Dimos Jones, Amy R. Powell, Richard A. Oberhelman

**Affiliations:** ^1^Tulane University School of Public Health and Tropical Medicine, New Orleans, LA, USA; ^2^Facultad de Salud Pública y Administración, Universidad Peruana Cayetano Heredia, Lima, Peru

**Keywords:** tuberculosis, delayed diagnosis, Latin America, qualitative research, HIV

## Abstract

**Introduction:** Tuberculosis (TB) remains a significant public health challenge worldwide, and particularly in Peru with one of the highest incidence rates in Latin America. TB patient behavior has a direct influence on whether a patient will receive timely diagnosis and successful treatment of their illness.

**Objectives:** The objective was to understand the complex factors that can impact TB patient health seeking behavior.

**Methods:** In-depth interviews were conducted with adult and parents of pediatric patients receiving TB treatment (*n* = 43), within that group a sub-group was also co-infected with HIV (*n* = 11).

**Results:** Almost all of the study participants recognized delays in seeking either their child’s or their own diagnosis of their TB symptoms. The principal reasons for treatment-seeking delays were lack of knowledge and confusion of TB symptoms, fear and embarrassment of receiving a TB diagnosis, and a patient tendency to self-medicate prior to seeking formal medical attention.

**Conclusion:** Health promotion activities that target patient delays have the potential to improve individual patient outcomes and mitigate the spread of TB at a community level.

## Introduction

Among communicable diseases, tuberculosis (TB) is the second leading cause of death worldwide, killing nearly two million people each year (1). Much of the global burden lies in the developing world, where certain region’s annual incidence can reach as high as 700 per 100,000 (2). Factors associated with poverty, such as malnutrition, overcrowding, poor ventilation, unemployment, and lack of access to healthcare prevent greater control of TB in many developing countries (3–8).

Peru has historically had one of the highest TB incidence rates in all of the Americas, with national incidence in 2011 estimated at 101/100,000 (9). In 1990, Peru implemented their National TB Program with a focus on the World Health Organization (WHO)-recommended Directly Observed Treatment Short-course (DOTS) strategy, and has since been heralded as the first high-burden TB country to successfully implement DOTS. Currently Peru’s National TB program covers the full treatment cost for TB patients, and in 2006 had reached a 78% treatment success rate (10). Despite the TB Program’s relative success, TB prevention and control remains a challenge in Peru. In 2009, USAID reported that although the population of Peru was only 3% of that of the Americas, it accounted for 12% of TB cases in the region (11). Peru is among the top eight countries in the world with the highest burden of multidrug-resistant TB (MDR-TB), with rates continuing to grow in recent years (12). The geographical distribution of cases of TB, MDR-TB, and most recently extremely drug-resistant TB (XDR-TB) are highly concentrated in the urban capital city of Lima when compared to the rest of Peru (13). Data reported in 2009 show that 2% of TB cases in Peru are also co-infected with HIV (10).

Efforts to curb the TB epidemic often focus on minimizing patient delays in seeking medical diagnosis of the disease. Due to the fact that most of TB transmission occurs between the onset of cough and the initiation of treatment (14), this period of patient delay is critical in mitigating disease transmission and minimizing the reproductive rate of the TB epidemic. It is generally considered that in order to achieve effective TB control that patient delay should not exceed more than 2 or 3 weeks (1). Effective case detection is critical to initiate timely treatment, however, in 2006 the World Health Organization estimated that only 70% of TB cases were being detected (10). It has been estimated that an infectious person who is not on treatment can infect between 10 and 15 people each year, and continue to become increasingly infectious as patient delay continues (14). Studies in both high and low income countries that have estimated time delays in obtaining diagnosis have found a significant time range – from 8 weeks in the United States to 12 weeks in Botswana and 26 weeks in Tanzania (15, 16).

In order to mitigate the TB epidemic, the reasons for patient delays in seeking a diagnosis and the complex individual and sociocultural factors that influence health seeking behaviors should be defined. Studies in developing countries have noted personal and psychosocial factors associated with delays in TB patients seeking care including HIV sero-status, gender, age, remote residence, alcohol consumption, low degree TB knowledge, perceived barriers to treatment, and fear of stigmatization (14, 17–20). Patients who seek care from informal health providers as an initial point of care or who self-medicate have also been associated with increased diagnostic delays (16, 21, 22). In general, the literature on TB diagnostic delay focuses on two main areas: (1) patient delay in seeking a health provider from the time of their first symptom and (2) health system delay, which refers to the healthcare facility’s capacity to produce relevant laboratory results and the healthcare providers’ efficacy of diagnosing the patient in a timely manner. The aims of this study are to explore patient’s perspectives around their barriers or delays associated with diagnosis seeking behavior for TB. The focus was only on self-reported reasons for delays in seeking diagnosis, no attempt to estimate the actual amount of delay was made, nor was there any attempt made to explore the diagnostic delays related to healthcare service provision.

## Materials and Methods

We describe the methods using the consolidated criteria for reporting qualitative research (COREQ) (23).

### Setting

This study took place in Lima, Peru. The city of Metropolitan Lima contains an estimated population 9,359,019 (24). Lima is divided into five main health regions: participants were recruited from five health centers and the regional hospital in the southern region of the city (DISA-South estimated population 2,105,296). The health facilities are located within the peri-urban neighborhoods of Villa Maria del Triunfo (estimated population 389,000), San Juan de Miraflores (estimated population 352,000), San Martin de Porres (579,000) and Villa El Salvador (411,000) (25, 26). These neighborhoods contain residents largely of low socio-economic status and inadequate sanitation. Previous research in the shantytowns on the outskirts of Lima showed TB hyperendemicity; 68% of adults in San Juan de Miraflores over the age of 25 were tuberculin positive (27).

### Data collection

Between 2004 and 2005, 43 in-depth interviews were conducted with adults who were receiving DOTS treatment for TB (*n* = 27) and parents of children who were receiving DOTS for TB (*n* = 16). The participants were in TB treatment for active TB, but were sputum negative, at recruitment. In the cases where the parent was interviewed for pediatric TB, most of the children interviewed were living with a parent with TB. Within the group, there were sub-groups of individuals who were co-infected with HIV (adults, *n* = 8; children, *n* = 3 – the three children were HIV infected through vertical transmission. Four similar interview guides were created and field tested during training (see below), one for each sub-population (TB, TB/HIV, parent of TB, parent of TB/HIV). All four interview guides included the same topics: interviewee description of the process of being diagnosed; their reasons for delays in seeking care; their general knowledge of TB (and HIV for TB/HIV cases); their feelings throughout the process of being diagnosed and receiving treatment; a description of the interactions they had or would have liked to have with health care providers, family and friends; what types of support helped them to continue their treatment (including physical and psychosocial support); and what suggestions they had for improving social support from family, friends, and providers (28). Findings associated to the impact of social support on TB patients within this study population were already published (28), and using the same data, this article focuses on patient’s perspectives on delays associated to seeking diagnosis. Specifically, questions regarding diagnostic delays were framed in two ways; (1) directly asking participants to explain the reasons for their own diagnostic delays; (2) indirectly asking participant for their point of view on why people in general might delay in seeking a diagnosis. Studies suggest that indirect questioning (IQ), allowing the respondent to not directly report on their own experience can provide more accurate information regarding sensitive behaviors which participants may be inclined to misrepresent (29). This IQ design was implemented in order to reduce levels of Social Desirability Bias, which commonly occurs when sensitive questions are asked and participants feel inclined to respond in a socially correct manner.

Participants were interviewed once, and interviews took approximately 60–90 min. The interviews were stopped when saturation was reached, the point at which no new themes or information emerged. This technique is commonly used in studies with non-probabilistic sampling. Audio recordings of each interview were transcribed after the lead investigator (Valerie A. Paz-Soldan) had listened to the interview. Any notes by either the research assistants or the lead investigator were incorporated into the transcripts. The lead investigator developed a code sheet derived from themes that were emerging in the interviews and based on the topics of interest; the code sheet was modified after the initial subset of ~20 interviews had been coded. Each interview was coded by two members of the research team. Inconsistencies in the coding were discussed by the team, and the lead investigator made a final call and used those moments to review the coding scheme with the two assistants. The coded transcripts were analyzed both manually and electronically using ATLAS.ti software.

### Research team

Two female research assistants were hired and trained for two weeks by the lead investigator, to conduct the 60–90 min long interviews with participants. Training consisted of reviewing the goals of the study and objective of each of the questions, practicing the interviews with the PI or with a volunteer while the PI observed and provided feedback. The research assistants were both psychologists working in the Peruvian National TB Program, in the northern cone of Lima (this research took place in the southern cone). The lead investigator (Valerie A. Paz-Soldan) is a Peruvian-American social scientist who was born and raised in Peru and currently lives there, has a wealth of qualitative research experience and experience at training and managing research teams. The lead investigator sat in on the eight initial interviews of each of the psychologists.

### Participants

Parents of TB and TB/HIV patients were recruited from the same health centers, as well as from the Instituto Nacional de Salud del Niño (the Peruvian Children’s Hospital). In Peru DOTS coverage is 100%, thus all participating TB patients were enrolled in DOTS therapy, which required that they be observed taking their TB medications by a healthcare professional at their health center. In each of these facilities, participants were recruited, using purposive or convenience sampling, via a clinician gatekeeper. The lead investigator and one of her research assistants explained the purpose of the research to the providers of the TB program. For reasons of patient confidentiality, the providers described the study to TB patients who met the inclusion criteria, and if the patients were interested in learning more, the provider referred them to the research assistant. The research assistant then described the study fully, and if the participant agreed to participate, a mutually convenient time and location was set for the interview. Most of the interviews took place in a private room within the health facility and some took place at the participant’s home or at another site. In all locations, the only ones present were the participant, or the parent and child, the research assistant, and in some cases, the lead investigator. Written informed consent for the interview and recording was obtained from all study participants prior to the interview.

### Reporting of results

Representative or interesting quotations were highlighted as we coded the text. Summaries of the main findings associated to delay were written up, and quotations relevant to the summaries were used to illustrate the themes. We also examined and reported any possible differences in our themes associated to sub-groups: gender, age groups, married vs. single, TB vs. TB/HIV, parent of TB or TB/HIV patient vs. TB or TB/HIV patient. We tried to obtain a range of participants; the only sub-groups that we stratified for ahead of time were by disease (TB vs. TB/HIV), and by age (adults vs. pediatrics). In the course of the coding, we started to observe differences between gender and partnership status.

### Ethical conduct of research

Prior to initiating this study, approval was obtained from the Institutional Review Boards of Tulane University School of Public Health and Tropical Medicine and from three local review boards: Peru’s Instituto Nacional de Salud del Niño, DISA-Sur authorities, and the Asociación Benéfica PRISMA (a local non-governmental organization).

## Results

Almost all of the study participants recognized delays in seeking either their child’s or their own diagnosis of their TB symptoms. Patient-related self-reported delays from our study population showed variable duration, spanning from 1 week to over 1 year before seeking formal medical attention after the onset of symptoms. Five principal reasons were identified from the data collected. This was done by thematically analyzing the data once all interviews had been completed. See Table 1.

**Table 1:** **Principal reasons for delays**.

Principal reason for delay	Details
Patient confusion about symptoms/lack of knowledge of TB	Participants: Did not seek healthcare because symptoms were not serious Were unaware of the range of symptoms
	For pediatric patients, parents:
	Took around 2 weeks before attending a clinic with a child with general symptoms Were unaware that children can contract TB
Self-medication	Participants: Used pharmaceutical or natural, homemade remedies Reported that informal healthcare is less expensive and less time consuming than visiting a clinic Reported waiting until symptoms were debilitating before seeking formal healthcare Reported self-medicating Reported self-medicating with pediatrics also
Work- related conflicts, including loss of time to be treated at a clinic	Participants: Reported work-related conflicts Reported long waiting times at clinics Reported that clinic times affect both formal and informal work hours
	Caregivers of pediatric patients: Cited work and long waiting times as reasons for delays Who had had TB themselves, were familiar with long waiting times and reported this as a reason for delaying taking their child to a clinic
Financial difficulties	Participants: Were not aware that TB treatment is free in Peru Reported that TB diagnostic tests are not free Reported that seeking medical care directly impacts purchasing of food for the family
Stigma: fear/embarrassment	Participants: Reported fear or embarrassment at the possibility of being diagnosed with TB Reported fear of discrimination
	Parents of children with TB: Reported fear that their children would be marginalized or rejected by friends at school, teachers, or neighbors.

### Symptom confusion

Symptoms such as chronic coughing, fever, and night sweats were frequently described as non- alarming occurrences that participants thought would improve over time and did not associate with TB infection. When one male participant showing febrile symptoms and weakness was asked if he initially suspected that he had TB, he responded:

No, I thought I had a simple fever, or something had not settled well with me, but I never imagined that!

Most patients reported that they had heard of TB and many were aware that it affected the lungs, however, they were not familiar with other symptoms. Numerous patients expressed confusion that their non-pulmonary symptoms, such as fevers or fatigue, were indicative of TB.

### Self-medication

Related to the common misunderstanding of TB symptomology, was the tendency for patients to initially treat their symptoms by self-medicating with pharmaceutical drugs or natural homemade remedies. A handful of patients also described consulting “naturalists” specializing in herbal remedies for diagnosis, and taking their recommended homemade products prior to seeking formal medical care. In most cases, pharmacists or naturalists provide what they feel to be helpful, effective advice. Participants tended to assume that their symptoms were not serious and could be treated with a basic drug from a pharmacy. An adult woman with TB explains:

Usually the pharmacy is easier for us because we don’t wait in a long line, or have to make an appointment… and afterward some feel better and never have to go to a clinic, others keep feeling sick and then they go to a clinic.

It was common for participants to not seek a diagnosis from medical providers until their symptoms were strong or debilitating. Four participants in particular, all adult males, described waiting until they had reached the point of having medical emergencies, such as fainting or coughing up blood, before being taken to hospital. A man, who did not seek care after coughing up blood for one week, describes why he waited:

Firstly, I did not know the symptoms of TB or that it was so deadly…And then I thought it [the bloody coughing] was just a little wound in my stomach or a small vein that had ruptured, or something like that which will cure itself quickly. I thought, oh its fine, nothing bad will happen.

### Work-related conflicts and loss of time

Taking time off work was the principal reason for approximately 20% of participants; either in terms of gaining permission or because of lost earnings. Many participants reported long lines at the health clinic, meaning they would have to arrive early and wait for several hours in order to receive attention.

A small proportion of participants who were not formally employed, but had other duties such as childcare or household responsibilities also noted time conflicts with visiting a health facility.

Parents of pediatric patients who had already been diagnosed as TB positive themselves were particularly familiar with enduring long waits at health facilities. One parent commented on her initial hesitation in taking her daughter to the hospital:

I didn’t want to go, I refused to go, and waiting for them to attend to me takes forever. When I had to go for myself they made me wait such a long time, maybe this is a reason why this time with my daughter I stayed home and didn’t go to the hospital with her…

### Although treatment is free, a diagnosis is not

The majority of participants expressed concerns about personal finances for themselves or others in their community. A small proportion of participants explained that if they had previously known that treatment was free, they would have not been as reluctant to seek care. Although all treatment costs are covered by the National TB Program, there are diagnostic tests (x-rays and laboratory tests) that need to be paid for by the patient prior to commencing treatment. Patients frequently described paying for diagnostic tests. One parent of a child with TB commented that

If you have many kids, you have to decide whether to take care of one or feed the rest.

Those who had sought care previously, and particularly those parents who had already been diagnosed with TB themselves, were already familiar with these charges, and were deterred to return to the health facility as a result. In addition, several respondents also described the process of enrolling themselves or their family members into the National TB Program to be cumbersome, confusing, and poorly supported by facility staff.

### Fear, embarrassment, and stigma

Another major theme that arose during the interviews was the prevalence of fear and embarrassment among participants and potential TB patients in the wider community. Only one fifth of study participants revealed that they themselves felt either fear or embarrassment regarding the possibility of being diagnosed with TB. Specifically, participants most commonly described fear and/or embarrassment resulting from either being seen by other members of their community in the health clinic, having a contagious disease, and being discriminated against by peers or health facility staff. Parents of children with TB reiterated the same feelings of fear that their children would be marginalized or rejected by friends at school, teachers, or neighbors. When asked to articulate his thoughts about patient healthcare-seeking delays in his community, one participant describes

They are scared of what people will say about them, we all suffer from this…one time or another someone will say to you, ‘you have a disease that affects people who don’t feed themselves well’… Now that I understand the disease better I know that anyone can get TB, but most people still see having tuberculosis as a taboo. They will point at you with their finger to say ‘you have TB’ and create this mistrust, then they will marginalize you.

### HIV/TB patients versus TB patients

There were no discernible differences when comparing the rationale for diagnosis delays given by HIV/TB patients versus TB patients. Both pointed to fear, embarrassment, and misunderstanding of the severity of their symptoms as principal reasons for their delays. Similar to TB patients, most HIV patients did not suspect that their symptoms were indicative of a serious illness like TB, causing the two groups to describe similar patient delay patterns. None of the HIV positive participants mentioned that they were aware of their increased risk for acquiring TB. Multiple HIV positive patients were initially misdiagnosed to have ailments such as stomach infection or a cold instead of TB.

### Hypothetical/indirect questioning

When the question asking “Why might someone chose to delay seeking medical diagnosis?,” was addressed to participants in the hypothetical sense (as opposed to using direct questioning; “Why did you delay in seeking a medical diagnosis?”), roughly half of the participants highlighted that someone in that position might feel scared or embarrassed of the consequences of their diagnosis. When compared to direct questioning results, roughly three times as many respondents mentioned fear or embarrassment as a possible reason for delayed test-seeking behavior when questioned indirectly. IQ also revealed a new response category, “ignorance,” which is a response that was not observed using direct questioning. When questioned indirectly, approximately one sixth of participants mentioned that a person’s ignorance may lead them to delay seeking medical diagnosis of their TB symptoms.

### Other

Although this paper does not intend to focus on healthcare service delays, a notable observation was that approximately one third of interviewees reported experiencing some form of medical misdiagnosis of their TB. Multiple participants reported being diagnosed and treated with medications for bronchitis, which was the most common misdiagnosis among this population. Other patients were reportedly misdiagnosed with pneumonia, pharyngitis, stomach infection, or flu. These misdiagnoses caused patients further delays before they received a correct diagnosis. One TB positive participant reported being given a sputum smear test, however, his results initially showed incorrectly that he was negative, leading him to treat his symptoms with cough syrup for 4 months until a different doctor suggested that he be retested. One 18-year-old woman with TB explained her process of being diagnosed with TB:

I had fever and a cough, so I went to the clinic and they did an analysis. It seemed that I had an ear infection, and they wanted to operate on my ear. Until after that, they did another analysis. I left there with nothing, that it was possibly a chronic sore throat. Then, I was given antibiotics, which was treatment for the sore throat, but after I completed the medication, the fever persisted. After that, I began to spit up blood. Then, I went to the health clinic and they told me that I had tuberculosis.

In a significant portion of pediatric cases, participants described longer and more complex diagnosis processes for their children, often visiting multiple healthcare institutions before receiving the proper diagnosis.

## Discussion

Almost all participants interviewed described delays in their decision to seek a formal medical diagnosis for their TB symptoms. This delay in health-seeking behavior is likely to contribute to increased risk of morbidity, mortality and TB transmission to other contacts in the community. It is essential to understand patient rationale behind their health-seeking decisions so that TB programs and health promotion messages can be constructed to target populations at high risk for diagnostic delays. There are five potential interventions that would address the main reasons for delaying seeking healthcare found in this study that we summarized in Table 2. These recommendations are for the peri-urban communities of southern Lima that are of a low socio-economic status. These strategies may not be applicable in other areas of Lima.

**Table 2:** **Potential interventions to improve health seeking behaviors**.

Potential intervention	Addressing which reason for delay
Enhance educational and health promotion interventions including improved training and support for pharmacists in recognition and management of TB and suspected TB	To improve public knowledge on range of TB symptoms Delivering health promotion through the well-used self-medication channels May help to reduce the social stigma
Improve ease of access to formal healthcare services by improving the efficiency of their client intake system	Limit self-medication Reduce patient wait times Increase attractiveness of health clinics Empowerment of patients to understand and navigate the national TB service
Create/improve infrastructure that supports patient privacy while waiting in line to receive TB diagnosis or treatment	May help to reduce the social stigma Encourage quicker health seeking behaviors
Further publicize the free treatment services being offered by the National TB Program	Mitigate patient diagnosis delays related to financial concerns.
Increased support services for pediatrics whose primary caregiver is being treated for TB	To help improve standard of care and outcomes

### TB symptoms can be ambiguous and confusing

The non-specific nature of TB symptoms is a core problem in TB case finding and diagnosis. Particularly in the early phases of the disease, symptoms are confounded with those of cold, malaria, and HIV. TB symptoms have also been commonly confused with symptoms for asthma and pneumonia (22, 30); a fact which is particularly relevant in Lima due to the high prevalence of acute respiratory illness in the region. The results from this study are in accordance with such findings; about half of our study participants did not connect their symptoms with the possibility of having TB.

Symptoms such as persistent cough, fevers, night sweats, and fatigue were described by participants as non-alarming, leading them to simply wait for the symptoms to pass on their own, or purchase medicine from a drug outlet or naturalist. The failure to connect their own symptoms with the possibility of having TB may be indicative of a lack of knowledge of the complete range of TB symptoms among the study population, as has been demonstrated in other studies in Peru (19, 20). Lack of knowledge of TB is likely to negatively affect healthcare seeking behavior, whilst those who are more knowledgeable are significantly associated with positive health-seeking behaviors (18). A study carried out in the Peruvian Amazon indicated that the general population’s understanding of TB symptomatology is poor; with only one fifth of the study population demonstrating understanding of airborne transmission through coughing (20).

Another element that may add to patient misunderstanding of respiratory symptoms in our study population is the high prevalence of acute respiratory infections (ARI) in Lima. In metropolitan Lima, 7.8% of children under the age of 5 suffer from symptoms of ARI (31). In the peri-urban regions surrounding Lima, rapid urbanization, air pollution, and the proliferation of dust contribute to Lima being the city with the highest incidence of ARIs in Peru (32). The persistence of respiratory symptoms among the general population, and the fact that many people with respiratory symptoms in Lima are in fact not infected with TB, may add to the confusion regarding when a cough is “just a normal cough” or whether a patient should indeed suspect TB. On a global scale, more than 95% of patients seeking treatment for chronic cough at a primary healthcare facility do not have TB (14). We suggest that the confusing nature of TB symptomatology be recognized and confronted in terms of patient education and health promotion messaging. Despite the fact that health education messages promoted in health facilities in Lima specifically encourage health seeking behaviors after 2 weeks of persistent cough, none of the respondents mentioned this time frame or reported having been exposed to these messages. Given the particular ambiguity of TB symptoms and their potential for confounding with other illnesses such as flu or other respiratory infections, it could be useful to enhance educational and health promotion interventions that encourage effective health-seeking behaviors in response to perceived TB symptoms. A study in Peru aimed at improving recognition and management of STI symptoms by providing additional training to pharmacists exhibited encouraging results (33).

### Culture of self-medication

One fourth of participants described medicating themselves or their children with products from either a drug outlet or homemade remedies to treat their symptoms prior to visiting a health clinic for a medical diagnosis. Previous research shows that prevalence rates for self-medication in Peru vary from 40 to 60% (34). A previous study documenting self-medication for TB symptoms in peri-urban Lima showed even higher rates of self-medication, where 63% of participants reported use of alternative remedies prior to presentation for investigation at a health center (21). A majority of those self-medicating in peri-urban Lima appear to be doing so through pharmacies, while a smaller proportion seek out traditional or herbal remedies. The consultation of “curanderos” (traditional healers), although popular in other areas in Peru, does not appear to be common among this urban population (21).

A previous study investigating self-medication rates in Peru showed that the most important factors influencing ones decision to self-medicate were (1) the patient’s access to healthcare (both physical and financial) or lack thereof, and (2) their relative access to medications (34). Results from our study echo those findings, as participants consistently expressed that their decision to self-treat was based on their perception that it would save time and money. Pharmacies in this peri-urban area are abundant, easy to access, do not have long queues and are able to offer options of varying price and strength.

### Practical constraints – work, time, money

Within our study population, preoccupation with work, time and money factored almost equally in terms of influencing participants reporting delays in seeking medical diagnoses. It is not surprising that among this economically disadvantaged population, earning an estimated annual median income of $2100 in 2000, compared to $7600 for the rest of the country (35) that such practical considerations were important factors guiding their health-seeking behavior. When participants expressed dismay at having to take time off work and the length of time required to wait for attendance at a health clinic, thus causing them to forego their regular income-generating activity (not because they are impatient). The large proportion of the working population in Peru does not have support from their employer or other government safety net to allow them to take time off of work without losing wages. An estimated 40% of the labor force in Peru is employed by small, informal micro-businesses, and only 20% have access to a formal pension plan (36). Both men and women voiced financial concerns, however, comments regarding preoccupation with work were slightly more common among men, which could be due to the dominance of traditional gender roles in the area. Statistics from 2011 show that in Lima women earn on average 61% of what men do (25). Despite widespread financial concerns among this population, multiple participants voiced that they had not been previously aware that TB treatment is offered for free by the National TB Program in Peru. Given the attention that participants in this study paid to voicing financial concerns related to missing work, making time-consuming visits to health centers, and paying for health services, one programmatic suggestion would be to further publicize the free treatment services being offered by the National TB Program in order to mitigate patient diagnosis delays related to financial concerns.

Our findings revealed that although the National TB Program offers free diagnostics and treatment to all patients presenting with TB symptoms, the reality is that these free services can be challenging for patients to access because the enrollment process is described as full of administrative paperwork and bureaucratic inefficiencies, which makes it difficult for patients to receive TB services for free, particularly their initial diagnostics. The interviews revealed that patients most commonly paid out of pocket (the out of pocket cost was not calculated) for diagnostics (sputum tests or X-rays) then by the time treatment was initiated, most patients had been able to enroll in the National TB Program and received their medications for free. An unfortunate reality in this community is that sometimes patients delay in presenting to a healthcare facility until they are very physically ill, and at that point they do not have the extra time and energy to put forth to the enrollment process. It seemed there was minimal patient support in terms of assistance with completing the enrollment paperwork, thus creating an additional barrier for those patients with low literacy or who are unfamiliar with the formal healthcare system. Most patients and their families do not have the time to enroll in the program due to child care responsibilities or work schedules (as previously discussed).

### TB vs. TB/HIV patient diagnosis delay

Research has shown that the diagnostic process for patients already diagnosed with HIV can be more complicated than it would be for regular TB patients, frequently resulting in delayed diagnosis (36). The diagnostic process of TB in HIV patients in resource limited settings, where the majority of HIV-TB cases occur, is complicated by the fact that immunosuppressed patients have an increased likelihood of smear negative disease (37) impeding early detection. Other research claims that the HIV tends to delay TB diagnosis due to the atypical laboratory and clinical presentation of TB in HIV-positive patients (15). However, there are other factors at play that have caused some researchers to raise the question of whether HIV might be “helpful” in speeding up diagnosis process for a patient co-infected with TB. It has been shown that the effects of antiretroviral therapy (ART) medications serve to unmask the subclinical symptoms of TB in HIV-infected patients, making their TB infection easier to detect (37). Other factors possibly contributing to more timely diagnosis in persons already infected with HIV are that (1) patients are already integrated participants in the healthcare system, (2) doctors should have a higher index of suspicion of TB infection given the patient’s increased risk, and (3) patient knowledge of TB risk and TB disease may increase as a result of their involvement in HIV care/support activities. Results from scientific studies on this topic offer conflicting results, and similarly, in this study, we found no discernible correlation between reported diagnostic delay and HIV seroprevalence. A recent study in rural South Africa showed that those patients who were already taking ARVs at the time of their TB diagnosis were 51% less likely to experience diagnostic delay (37). A study performed by the WHO in Syria found HIV seroprevalence to be a risk factor for diagnostic delay, however, other studies in Ghana and Thailand have concluded the opposite (14).

### Comments on guide design – allowing for hypothetical responses

As described in the methods section, when asked about delays associated to seeking diagnostic care, we asked both about their own experience (direct) and as a hypothetical situation (i.e., “why might people delay in seeking care…,” indirect). We observed that the content of these interview questions certainly raised emotional responses by many respondents recounting the traumas of their illness and their regrets for delaying the diagnosis process. Responses from participants answering to IQ tended to differ significantly from when questioned directly about their experience; direct questions regarding self-reported diagnostic delay tended to describe more practical and “socially acceptable” reasons to explain their diagnostic delay, such as having to work or not having the money to attend a clinic. Responses to indirect (third person) questioning evoked more personal responses about delicate social issues such as “ignorance,” “fear,” “embarrassment,” and “negligence,” which are characteristics that respondents may have been uncomfortable admitting about themselves if they had been asked directly. In fact, when comparing our findings from direct questioning and IQ, we found that when IQ was used, about three times as many participants spoke about elements of social stigma (fear and embarrassment) as a possible reason for diagnostic delay.

In the context of this study, we found that utilizing IQ for sensitive topics served to extract richer, more detailed information from participants than they may have been willing to give using standard direct questioning, and would recommend the use of this technique in future qualitative research. Given the apparent social sensitivity surrounding TB diagnosis in this population, one programmatic implication could be the provision of more private spaces for client intake and testing. Clinic infrastructure that supports patient privacy while waiting in line to receive TB diagnosis or treatment may help to reduce the social stigma experienced by patients, – but it could also act in the opposite manner by implying that patients should hide. It may be key to explore the options that might be available in health facilities in Peru, and conduct qualitative research with TB patients and providers to determine which is the best fit for this population.

### Delays in treatment for pediatric patients

Among parents of pediatric patients, reported health system delays were brought up more often than these diagnostic delays among adult patients. These delays included misdiagnosis and parents being sent to multiple institutions before having their child attended by a facility that was properly equipped to test the child for TB. Although children comprise a significant portion of TB infections, TB diagnosis in children is more complicated and difficult to carry out with accuracy due to the lack of sputum production in pediatric patients (38). In less developed countries such as Peru, the best diagnostic technologies that would help to overcome this barrier are too expensive for most, thus diagnostic methods for pediatric TB vary depending on the available resources at the healthcare facility (38, 39). The risk of clinical error in pediatric TB diagnosis is high; a study suggests that the burden of childhood TB within a population could be under or over estimated by up to 82% using structured diagnostic systems (40). Diagnostic difficulties associated with pediatric TB could be contributing to overall diagnostic delays and thus an increased period of infectiousness in pediatric patients.

Another issue that arose among our pediatric patient population was the fact that a significant proportion of them were being cared for by parents who were also suffering from TB, many having been infected from their mothers through vertical transmission. Chemotherapy for TB commonly causes fatigue, nausea, and other debilitating side effects. Mothers or other caregivers who are being treated for TB are likely not in proper physical condition to be caring for a child with TB. Pediatric patients in this situation should be considered at high-risk for poor treatment outcomes, and programs should consider what types of support services can be made available to facilitate access to care for this vulnerable population.

### In comparison to similar studies

Other studies conducted in Nigeria and Peru found similar evidence regarding reasons for their delays (20, 41). In Nigeria, delays in seeking care were positively associated with poor knowledge of TB symptoms (41). However, this study using structured questionnaires did not look at stigma or fear of discrimination as possible reasons why there are delays in health seeking behaviors. In another study in Peru, they found that nutritional incentives were the main reason for treatment adherence, and a motivator to seek treatment (20). Only one of their interviewees (out of 73) reported stigmatized, and it was not brought up as a barrier to health care seeking, but this may have been because IQ was not employed.

### Limitations

Our findings capture only certain issues and trends from a limited sample size of adult and pediatric patients of TB and TB/HIV, and are thus not intended to be representative of the general population. There was clear selection bias due to the fact that all participants were recruited through a healthcare provider, were currently receiving treatment for their TB, and all agreed to speak to an interviewer about their specific condition. In order to protect our interviewers from potential TB exposure, we did not speak with any patients who had stopped receiving treatment, which also contributes to the selection bias. It was particularly difficult to find pediatric HIV/TB patients within our study area, thus this sub-population was underrepresented within our study population. As this study intended to capture the patient perspective and their healthcare seeking motivations, and was all self-reported retrospectively, no quantitative information regarding actual delay time was collected for analysis.

## Conclusion

Health promotion activities that target patient delays have the potential to improve individual patient outcomes and mitigate the spread of TB at a community level. Principal explanations for patient delay in this study population were (1) lack of knowledge and understanding of the potential severity of their symptoms as they related to TB; (2) self-medication with pharmaceutical or natural remedies to treat symptoms; (3) work-related conflicts, lack of available time to visit a health clinic; (4) financial concerns about paying for a visit to a health clinic; and (5) presence of social stigma in the community. Although not admitted by many participants in direct questioning about their own personal TB diagnosis experience, IQ allowing participants to respond in the third person revealed high recognition of fear, embarrassment related to the presence of social stigma of TB in this study population, which may have resulted in negative healthcare-seeking behavior. Programmatic improvements for consideration include enhanced educational and health promotion intervention including support for pharmacists; increased publicizing of free TB treatment offered by the National TB Program; improve healthcare facility efficiency to reduce patient wait times; improved infrastructure to support patient privacy; facilitate supportive care for pediatric patients whose caregivers are also ill with TB.

## Conflict of Interest Statement

The authors declare that the research was conducted in the absence of any commercial or financial relationships that could be construed as a potential conflict of interest.
